# Acclimation and stress response of *Prochlorococcus* to low salinity

**DOI:** 10.3389/fmicb.2022.1038136

**Published:** 2022-10-13

**Authors:** Xiayu He, Huan Liu, Lijuan Long, Junde Dong, Sijun Huang

**Affiliations:** ^1^Key Laboratory of Tropical Marine Bio-resources and Ecology, South China Sea Institute of Oceanology, Chinese Academy of Sciences, Guangzhou, China; ^2^College of Earth and Planetary Sciences, University of Chinese Academy of Sciences, Huairou, Beijing, China; ^3^Southern Marine Science and Engineering Guangdong Laboratory (Guangzhou), Guangzhou, China

**Keywords:** *Prochlorococcus*, transcriptome, low salinity acclimation, low salinity stress, RNAseq

## Abstract

*Prochlorococcus* is an obligate marine microorganism and the dominant autotroph in tropical and subtropical open ocean. However, the salinity range for growing and response to low salinity exposure of *Prochlorococcus* are still unknown. In this study, we found that low-light adapted *Prochlorococcus* stain NATL1A and high-light adapted strain MED4 could be acclimated in the lowest salinity of 25 and 28 psu, respectively. Analysis of the effective quantum yield of PSII photochemistry (F_v_/F_m_) indicated that both strains were stressed when growing in salinity lower than 34 psu. We then compared the global transcriptome of low salinity (28 psu) acclimated cells and cells growing in normal seawater salinity (34 psu). The transcriptomic responses of NATL1A and MED4 were approximately different, with more differentially expressed genes in NATL1A (525 genes) than in MED4 (277 genes). To cope with low salinity, NATL1A down-regulated the transcript of genes involved in translation, ribosomal structure and biogenesis and ATP-production, and up-regulated photosynthesis-related genes, while MED4 regulated these genes in an opposite way. In addition, both strains up-regulated an iron ABC transporter gene, *idiA*, suggesting low salinity acclimated cells could be iron limited. This study demonstrated the growing salinity range of *Prochlorococcus* cells and their global gene expression changes due to low salinity stress.

## Introduction

Cyanobacterium *Prochlorococcus* is the smallest and most abundant photosynthetic, oxygen-evolving organism on Earth, playing a significant role in carbon fixation and biogeochemical cycles in the ocean (Guillard et al., 1985; Goericke and Welschmeyer, 1993; Liu et al., 1997). The prokaryotic *Prochlorococcus* cells contain divinyl-chlorophyll a and both monovinyl and divinyl-chlorophyll b as their primary photosynthetic pigments, which are unique to other cyanobacteria that contain chlorophyll a and phycobiliprotein (Chisholm et al., 1992; Hess et al., 1996). *Prochlorococcus* is believed to be an obligate marine organism that is predominantly found in oligotrophic open oceans, as well as in some coastal waters, but barely seen in low salinity estuarine waters (Flombaum et al., 2013). *Prochlorococcus* thrives throughout the euphotic zone in the tropical and subtropical oceans from 45° N to 40° S (Scanlan et al., 2009). This genus of marine picocyanobacteria is divided into high-light (HL) adapted and low-light (LL) adapted ecotypes, which are also phylogenetically distinct (Ferris and Palenik, 1998; Moore and Chisholm, 1999). HL ecotypes are usually distributed in upper euphotic zone, while LL ecotypes are generally distributed in the lower to bottom euphotic zone (Johnson et al., 2006; Zinser et al., 2007). Besides the light-related niche partitioning of HL and LL ecotypes, two HL ecotypes, HLI and HLII, also display temperature-related niche partitioning that HLII ecotypes dominate the warmer oceans between 30° N and 30° S while HLI ecotypes dominate the higher latitude oceans (West et al., 2001; Rocap et al., 2003; Mühling, 2012; Voigt et al., 2014). Despite comprising diverse phylogenetic lineages, *Prochlorococcus* is monophyletic on the phylogenetic tree built on 16S rRNA sequences of cyanobacteria (Rocap et al., 2002). *Synechococcus* is the sister genus of *Prochlorococcus*. However, *Synechococcus* is a provisional genus containing polyphyletic clusters which are scattering on the phylogenetic tree of cyanobacteria (Robertson et al., 2001). Marine *Synechococcus* is affiliated with cluster 5, which comprises subclusters 5.1, 5.2 and 5.3. In contrast to *Prochlorococcus*, marine *Synechococcus* is much more widely distributed, existing from estuary to open ocean and from equatorial to polar regions (Partensky et al., 1999; Zwirglmaier et al., 2008).

Salinity is a crucial factor affecting the growth and biogeography of cyanobacteria (Scanlan et al., 2009). There were plenty of studies on cyanobacteria’s salt acclimation and salt stress response (Hagemann, 2011). However, most of those studies were conducted mainly on freshwater cyanobacteria such as the euryhaline *Synechococcus* strain PCC 7002 and moderately halotolerant *Synechocystis* strain PCC 6803 rather than typical marine cyanobacteria such as *Prochlorococcus* or marine *Synechococcus* (Hagemann, 2011). For example, when growing at high salinity, *Synechococcus* PCC 7002 had increased expression of genes involved in compatible solute biosynthesis and electron transport, while only minor changes were observed when cells were grown at low salinity (Ludwig and Bryant, 2012). It also has been revealed that 200–300 genes were up-regulated and a comparable number of genes were down-regulated after the addition of salt in *Synechocystis* PCC 6803 (Kanesaki et al., 2002; Marin et al., 2003). Secondly, very few studies focus on the acclimation and stress response of marine cyanobacteria to low salinity. Lastly, compared to *Synechococcus*, salinity-related physiological studies on *Prochlorococcus* are even more seldom. A recent study showed that *Prochlorococcus* strain AS9601 could be acclimated to a high salt concentration of 5% (w/v; Al-Hosani et al., 2015). The authors compared the growth rate and transcriptome of AS9601 at salinities 3.8% (w/v) and 5% (w/v), and found that, under high salt concentration, approximately one-third of the genome expressed differentially.

The strict biogeographic distribution of *Prochlorococcus* in oceanic waters suggests that this organism cannot be adapted to low salinity. However, what is the lowest salinity that *Prochlorococcus* can survive and what is the stress response of *Prochlorococcus* cells to low salinity are still unclear. In this study we first tested the salinity range of two *Prochlorococcus* strains, NATL1A and MED4, and then acclimatized the two strains under different salinities. We found that the lowest acclimation salinity is 25 psu for MED4 and 28 psu for NATL1A. Both NATL1A and MED4 cells were stressed when growing in salinities lower than 34 psu. We also found that the transcriptomic response of the two strains to low salinity stress were highly different.

## Materials and methods

### Strains and growth conditions

*Prochlorococcus* strains MED4 and NATL1A were obtained from Jiao Nianzhi Lab, Xiamen University. Cultures were maintained in Pro99 natural seawater medium with a salinity of 34 psu, at 21°C and under a constant light intensity of 10 μE m^−2^ s^−1^. We used canted neck polystyrene flasks (Corning Inc., Corning, NY, United States) of different volumes to culture the *Prochlorococcus* strains.

### Experiment setup and growth rate calculation

Preparation of Pro99 medium followed the protocol from the Chisholm Laboratory.^1^ The seawater from the South China Sea basin was filtered through 0.22 μm polycarbonate membrane, and the salinity was pre-adjusted to 22 psu ~ 60 psu with a 2 psu interval using ddH_2_O or NaCl. Salinity was measured using an ATAGO PAL-06S refractometer (ATAGO, Japan). These seawaters were autoclaved at 121°C for 15 min. Macronutrient (NH_4_Cl and NaH_2_PO_4_) stocks and the trace metal stock were prepared in advance, and they were added into the above seawater base. *Prochlorococcus* cultures growing in the Pro99 medium of salinity 34 psu were inoculated into the salinity gradient mediums. The salinity was finally adjusted to 22 psu ~ 60 psu using the ddH_2_O with Pro99 nutrients. *Prochlorococcus* growth was monitored every day for 2 weeks by measuring the OD440 absorbance using a multimode plate reader (PerkinElmer, Waltham, MA, United States) and measuring the cell abundance using a flow cytometer (BD Accuri C6, BD Biosciences, CA, United States). Three biological replicates were set up for the experiment. Growth rate was calculated based on the two monitoring methods, respectively. Growth rate was calculated according to Mackey et al. (2013): *T*_d_ = Ln (N_i + 1_/N_i_), N_i + 1_ is the number of cells on day i + 1, N_i_ is the number of cells on day i, *T*_d_ is the growth rate of cells. The average growth rate of cells was calculated during the logarithmic phase.

### Low salinity acclimation

*Prochlorococcus* strains MED4 and NATL1A were acclimated to different salinities (24 psu, 25 psu, 26 psu, 27 psu, 28 psu, 30 psu, 32 psu, 34 psu) by consecutive transfers from exponential growing cultures to fresh media. Three biological replicates were set up for each salinity. Five rounds of transfer were conducted for each strain. Using flow cytometry, cell abundance was monitored at day 0, day 5 and day 10. To assess the stress to low salinity, each strain’s dark-adapted photochemical efficiency (F_v_/F_m_) was monitored on day 10 in each round, using a handheld fluorometer (AquaPen AP 110/C, Photon Systems Instruments). To measure F_v_/F_m_, 1 ml culture was dark-adapted in the sample cuvette for 15–30 min. The maximal fluorescence levels (F_m_) were measured in the dark and under bright purple light (455 nm, 100 μEm^−2^ s^−1^), where F_0_ is the basal fluorescence level and F_v_ is the variable fluorescence. The PSII quantum yield was calculated as F_v_/F_m_ = (F_m_–F_0_)/F_m_.

### RNAseq analysis

To acclimate the *Prochlorococcus* strains, MED4 and NATL1A were growing in the Pro99 medium of salinity 28 psu and 34 psu for five rounds of inoculation. Then the acclimated cultures were inoculated in fresh medium of salinity 28 psu and 34 psu, with salinity 34 psu being the control. Three biological replicates were set up. During the exponential growth phase, 100 ml cultures were filtered onto 0.22 μm polycarbonate membrane to collect cells and the membranes were immediately flash frozen in RNAlater by liquid nitrogen and stored at −80°C until RNA extraction. Total RNA was extracted from the membrane using the MagZol Reagent (Magen Biotech, Guangzhou, China). Sequencing libraries were prepared using VAHTS™ Stranded mRNA-seq Library Prep Kit for Illumina® (Vazyme biotech co., Ltd., Nanjing, China) following the manufacturer’s instructions. Libraries were multiplexed and sequencing was carried out on an Illumina HiSeq system with the 2 × 150 paired-end (PE) configuration (GENEWIZ). Cutadapt (v1. 9. 1) was used to remove adapters, primers, and reads with a base quality <20 based on FASTQ files. Clean data were aligned to the MED4 and NATL1A genomes *via* Bowtie2 software (v2. 1. 0). HTSeq (v0. 6. 1p1) was used to estimate gene expression levels from clean data. Differential expression analysis was performed using the DESeq Bioconductor package, a model based on negative binomial distribution. After adjusting using Benjamini and Hochberg’s approach for controlling the false discovery rate, differentially expressed genes were considered significant at value of *p* < 0.05. Highly induced or suppressed genes were considered as meeting both false discovery rate *p* < 0.05 and magnitude of log2fold change with values greater than 1 (highly induced) or less than −1 (highly suppressed). These two different criterions were also used in a previous study (Al-Hosani et al., 2015). Transcriptomic data have been deposited in NCBI Gene Expression Omnibus (GEO) under the accession number GSE195946.

## Results and discussion

### Salinity range and acclimation to low salinity of *Prochlorococcus*

**Figure 1:**
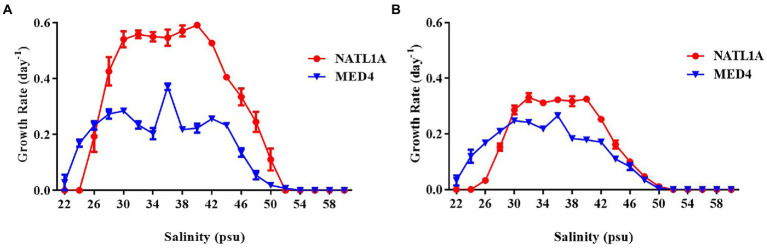
Growth rate of *Prochlorococcus* strains NATL1A and MED4 growing in Pro99 medium with salinities from 22 psu to 60 psu. Flow cytometry **(A)** and absorbance (OD440) measurement of chlorophyll **(B)** were used to monitor growth.

High-light adapted *Prochlorococcus* strain MED4 and low-light adapted strain NATL1A were tested for growth in different salinities ranging from 22 psu to 60 psu. Cell counting through flow cytometry (Figure 1A) and absorbance measurement at 440 nm (Figure 1B) were used to monitor the growth of *Prochlorococcus* cells. MED4 could grow in the salinity range from 22 psu to 50 psu and NATL1A could grow in the range from 26 psu to 50 psu. The optimal salinity ranges of MED4 and NATL1A were similar, from 30 psu to 40 psu. This result came from the first transfer of cultures from salinity 34 psu to other salinities. Under the same growing temperature (21°C) and light intensity (10 μE m^−2^ s^−1^), the LL strain NATL1A grew faster than the HL strain MED4.

**Figure 2:**
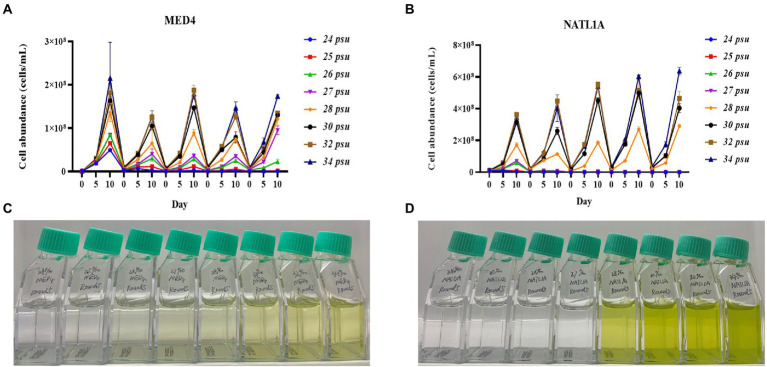
Acclimation of *Prochlorococcus* strains NATL1A and MED4 in different salinities. Five rounds of transfers were carried out and the growth **(A,B)** were monitored by flow cytometry. The pictures **(C,D)** showed the last round of cultures.

**Figure 3:**
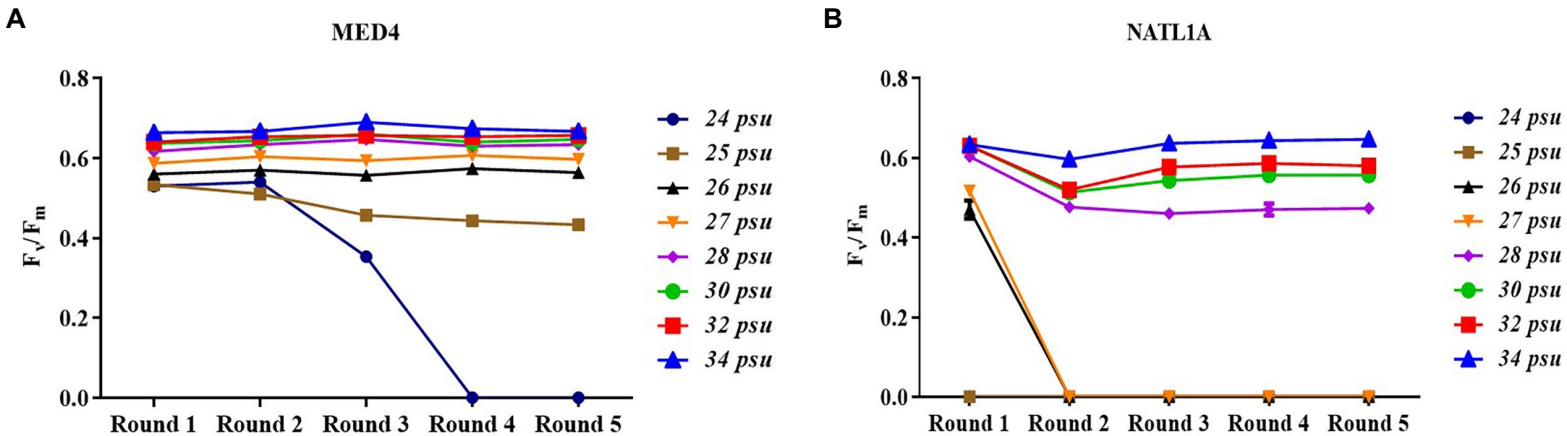
Dark-adapted photochemical efficiency (Fv/Fm) of *Prochlorococcus* strains MED4 **(A)** and NATL1A **(B)** growing in different salinities during acclimation.

During the acclimation experiment, NATL1A could grow in salinities higher than 26 psu in the first round, but could not survive in salinity lower than 28 psu in the last round (Figure 2B). Interestingly, MED4 showed a gradually changing growth rate in the salinity gradient from 25 psu to 28 psu, while NATL1A showed a sharp change between salinity 27 psu and salinity 28 psu (Figures 2C,D). The effective quantum yield of PSII photochemistry (F_v_/F_m_) was measured on the 10th day at the end of each incubation round (Figure 3). Both strains showed reduced yield when growing in low salinities from 24 psu to 32 psu, compared to the yield when growing in salinity 34 psu, and the lower the salinity resulted in lower yield. The yield of MED4 growing in salinity 24 psu was not detectable after round 4, while the yield of NATL1A growing in salinities 27 psu and below was not detectable after round 2. Together, these data showed that *Prochlorococcus* MED4 and NATL1A could be acclimated in salinities 25 psu and 28 psu, respectively. Interesting, the high-light adapted strain MED4 and low-light adapted strain NATL1A showed different tolerance to low salinity.

It is well known that *Prochlorococcus* is an oceanic microorganism (Partensky et al., 1999), although a few studies claimed that *Prochlorococcus*-like populations existed in estuarine and even freshwater environments (Corzo et al., 1999; Shang et al., 2007; Mitbavkar et al., 2012; Zhang et al., 2013). However, these studies all only depended on flow cytometry investigation, and could not confirm that those “populations” on the flow cytometry diagram were indeed *Prochlorococcus*. Our acclimation study suggests that *Prochlorococcus* cannot live in salinity lower than 25 psu for a long time period (50 days in this study). This study provides evidence supporting that *Prochlorococcus* is an oceanic organism.

### Differentially expressed genes in low salinity acclimated *Prochlorococcus* cells

RNA-seq was performed to assess the response of acclimated *Prochlorococcus* cells to low salinity (28 psu), with the salinity 34 psu being the control. When the filter criteria of significance meet the *value of p* < 0.05, there were 525 differentially expressed genes in the low salinity acclimated cells of NATL1A, with 286 genes being induced and 239 genes being repressed (Table 1). By contrast, MED4 appears to be less fluctuant under low salinity stress, with only 277 differentially expressed genes, among which 146 were induced and 131 were repressed (Table 1). A previous study compared the transcriptomes of *Prochlorococcus* AS9601 under high salt stress (5.0%) and under normal salt concentration (3.8%), and found 627 differentially expressed genes (Al-Hosani et al., 2015). Together, these results suggest that *Prochlorococcus* is sensitive to salinity changes.

**Table 1:** Functional categorization of differentially expressed genes (*p* < 0.05) in low salinity acclimated cells of NATL1A and MED4.

Function categories	Total no.		Total no. differential expressed		No. induced		No. repressed		Prevalent expression profile	
	NATL1A	MED4	NATL1A	MED4	NATL1A	MED4	NATL1A	MED4	NATL1A	MED4
Translation, ribosomal structure and biogenesis	128	130	38	25	5	24	33	1	Repressed	Induced
Transcription	26	27	10	6	7	5	3	1	Induced	Induced
Signal transduction mechanisms	26	24	7	3	3	2	4	1	Equal	Equal
Secondary metabolites biosynthesis, transport, and catabolism	18	22	4	7	3	2	1	5	Equal	Repressed
Replication, recombination, and repair	4	4	2	1	2	1	0	0	Equal	Equal
Posttranslational modification, protein turnover, chaperones	87	82	23	19	13	5	10	14	Induced	Repressed
Nucleotide transport and metabolism	48	50	14	5	8	4	6	1	Equal	Induced
Lipid metabolism	29	31	10	6	3	3	7	3	Repressed	Equal
Intracellular trafficking and secretion	14	12	4	2	2	1	2	1	Equal	Equal
Inorganic ion transport and metabolism	58	57	8	6	5	2	3	4	Equal	Equal
Energy production and conversion	77	78	28	14	19	8	9	6	Induced	Equal
DNA replication, recombination, and repair	68	65	12	7	7	5	5	2	Equal	Induced
Defense mechanisms	17	16	2	1	2	0	0	1	Equal	Equal
Coenzyme metabolism	105	101	20	11	6	4	14	7	Repressed	Repressed
Cell wall/ membrane/envelope biogenesis	1	2	1	1	1	0	0	1	Equal	Equal
Cell envelope biogenesis, outer membrane	93	91	30	10	16	6	14	4	Equal	Equal
Cell division and chromosome partitioning	14	16	4	3	3	1	1	2	Equal	Equal
Cell cycle control, cell division, chromosome partitioning	2	2	0	1	0	1	0	0	Equal	Equal
Carbohydrate transport and metabolism	46	48	10	10	4	6	6	4	Equal	Equal
Amino acid transport and metabolism	128	118	38	17	19	11	19	6	Equal	Induced
Function unknown	86	88	18	13	10	4	8	9	–	–
General function prediction only	137	132	44	13	23	8	21	5	–	–
Others	6	5	2	0	0	0	2	0	–	–
Not in COGs	1,021	841	196	96	125	43	71	53	–	–
Total	2,239	2042	525	277	286	146	239	131	–	–

Subsequently, the differentially expressed genes of these two strains were functionally classified according to Cyanobase definitions (Fujisawa et al., 2014). Firstly, the numbers of induced and repressed genes were equal for most functional modules (60–70%) in each of the two strains. Secondly, compared to the control group, the changed prevalent expression profiles between the two strains were different. In low salinity acclimated NATL1A, some genes involved in translation, ribosomal structure and biogenesis, lipid metabolism and coenzyme metabolism were down-regulated, while genes involved in transcription, posttranslational modification, protein turnover, chaperones and energy production and conversion were up-regulated (Table 1). However, in MED4, regulation profile of the functions mentioned above is different from NATL1A, except for coenzyme metabolism.

### Contrasting regulation between NATL1A and MED4

Most interestingly, among the genes involved in translation, ribosomal structure and biogenesis, five were up-regulated and 33 were down-regulated in low salinity acclimated NATL1A cells compared to control (Tables 1, 2). However, in MED4, 24 genes of those genes were up-regulated and only one was down-regulated (Tables 1, 3). Strikingly, the regulation of genes involved in energy production and conversion were also in distinct patterns between NATL1A and MED4. In low salinity acclimated NATL1A cells, the ATP-producing genes were down-regulated (*atpA*, *atpC*, *atpD*, *atpH* and other ATP synthase genes), while many genes involved in photosynthesis (*psaC*, *psb27*, *rbcS*), cytochrome oxidation (*cyoA*, *cyoB*, *ctaE*), NADH dehydrogenase (*ndhA*, *ndhH*) were up-regulated (Table 2). However, in low salinity acclimated MED4 cells, genes for photosynthesis were down-regulated, such as photosystems II (*psbA*, *psbB*, *psbD*, *psbN*), cytochrome F (*petA*), and electron transport chain intermediate (*ndhD*), while most ATP-producing genes (*acnB*, *atpG*, *atpF*, *atpH*, *atpD*) were up-regulated. This striking contrasting transcriptional regulation indicated the two strains processed different response mechanisms to low salinity stress. It is likely that, to respond to low salinity stress, NATL1A enhanced photosynthesis but repressed ATP production and translation and biosynthesis. In contrast, MED4 repressed photosynthesis but enhanced ATP production, translation and biosynthesis. The reason is possible that NATL1A and MED4 were in different stress level under the salinity 28 psu, which appears to be slightly stressful for MED4, but extremely stressful for NATL1A. This is the reason why the differentially expressed genes of NATL1A were more than those of MED4.

**Table 2:** List of a part of differentially expressed genes (*p* < 0.05) in low salinity acclimated *Prochlorococcus* NATL1A.

Gene ID	Gene name	Product	*p-*Value	log_2_FC
*Energy production and conversion*				
gene-NATL1_05001	*cyoA*	putative cytochrome c oxidase, subunit 2	< 0.001	1.249
gene-NATL1_17081	*acoA*	Pyruvate dehydrogenase E1 alpha subunit	< 0.001	0.979
gene-NATL1_04991	*cyoB*	Cytochrome c oxidase, subunit I	< 0.001	0.897
gene-NATL1_06051	*rbcS*	Ribulose bisphosphate carboxylase, small chain	0.010	0.780
gene-NATL1_02471	*ndhH*	putative NADH dehydrogenase subunit	< 0.001	0.770
gene-NATL1_20041	NATL1_20041	NADH dehydrogenase I subunit N	< 0.001	0.723
gene-NATL1_05651	*psb27*	possible Photosystem II reaction center Psb27 protein	< 0.001	0.6925
gene-NATL1_05981	*chlN*	Light-independent protochlorophyllide reductase subunit N	< 0.001	0.650
gene-NATL1_20591	*psaC*	Photosystem I subunit PsaC	0.004	0.633
gene-NATL1_04561	*pdhC*	Dihydrolipoamide acetyltransferase	0.001	0.607
gene-NATL1_04981	*ctaE*	Cytochrome c oxidase, subunit III	0.002	0.580
gene-NATL1_20451	*icd*	Isocitrate dehydrogenase	0.002	0.547
gene-NATL1_04171	*petB*	Cytochrome b6	0.006	0.532
gene-NATL1_02331	*ndhA*	putative respiratory-chain NADH dehydrogenase subunit	0.006	0.498
gene-NATL1_17231	NATL1_17231	FAD/FMN-containing dehydrogenases	0.024	0.484
gene-NATL1_03751	*rub*	probable rubredoxin	0.0158	0.453
gene-NATL1_21811	*acnB*	Aconitate hydratase B	0.0188	0.394
gene-NATL1_03311	*psbI*	photosystem II reaction center PsbI protein	0.0263	−0.446
gene-NATL1_19381	NATL1_19381	Fe-S oxidoreductase	0.021	−0.482
gene-NATL1_18501	*atpH*	ATP synthase, delta (OSCP) subunit	0.008	−0.497
gene-NATL1_18491	*atpA*	ATP synthase F1, alpha subunit	0.006	−0.565
gene-NATL1_19601	*psaI*	photosystem I subunit VIII (PsaI)	0.008	−0.574
gene-NATL1_00561	NATL1_00561	Flavoprotein, FldA	0.009	−0.647
gene-NATL1_18481	NATL1_18481	ATP synthase gamma subunit	< 0.001	−0.668
gene-NATL1_18511	NATL1_18511	ATP synthase B/B′ *CF*(0)	< 0.001	−0.671
gene-NATL1_18381	*atpD*	ATP synthase F1, beta subunit	< 0.001	−0.723
gene-NATL1_14931	*gldA*	putative glycerol dehydrogenase	0.004	−0.755
gene-NATL1_18391	*atpC*	ATP synthase, Epsilon subunit	< 0.001	−1.089
*Inorganic ion transport and metabolism*				
gene-NATL1_16181	*afuA*	putative iron ABC transporter, substrate binding protein	< 0.001	1.208
gene-NATL1_19031	NATL1_19031	Ferric uptake regulator family	< 0.001	0.853
gene-NATL1_05281	*nhaP*	putative Na^+^/H^+^ antiporter, CPA1 family	0.005	0.597
gene-NATL1_20831	*mgtE*	MgtE family, putative magnesium transport protein	0.009	0.501
gene-NATL1_03411	*amtB*	Ammonium transporter family	0.022	0.414
gene-NATL1_03071	*met3*	ATP-sulfurylase	0.002	−0.582
gene-NATL1_15081	*petH*	ferredoxin-NADP oxidoreductase (FNR)	< 0.001	−0.687
*Molecular chaperone*				
gene-NATL1_09851	NATL1_09851	Molecular chaperone DnaK, heat shock protein hsp70	0.001	0.668
gene-NATL1_21861	NATL1_21861	Molecular chaperone DnaK2, heat shock protein hsp70-2	0.010	0.624
*Translation, ribosomal structure and biogenesis*				
gene-NATL1_18631	NATL1_18631	FtsJ cell division protein: S4 domain:Hemolysin A	0.001	0.837
gene-NATL1_04781	NATL1_04781	tRNA/rRNA methyltransferase (SpoU)	0.006	0.573
gene-NATL1_00131	NATL1_00131	tRNA-dihydrouridine synthase	0.040	0.566
gene-NATL1_04521	*lrtA*	light repressed protein A-like protein	0.002	0.563
gene-NATL1_03171	*ileS*	Isoleucyl-tRNA synthetase	0.016	0.425
gene-NATL1_04021	*rps1a*	30S ribosomal protein S1, protein A	0.036	−0.379
gene-NATL1_17711	*rplU*	50S ribosomal protein L21	0.025	−0.445
gene-NATL1_16641	*rpsN*	30S Ribosomal protein S14	0.011	−0.455
gene-NATL1_05781	*frr*	Ribosome recycling factor	0.022	−0.457
gene-NATL1_19971	*rpsC*	30S ribosomal protein S3	0.016	−0.480
gene-NATL1_07951	*glyS*	Glycyl-tRNA synthetase beta subunit	0.013	−0.485
gene-NATL1_19921	*rplX*	50S ribosomal protein L24	0.022	−0.490
gene-NATL1_07891	*rpsB*	30S ribosomal protein S2	0.015	−0.497
gene-NATL1_02771	*rplL*	50S ribosomal protein L7/L12	0.004	−0.564
gene-NATL1_19481	*rpsJ*	30S ribosomal protein S10	0.001	−0.568
gene-NATL1_21621	*aspS*	Aspartyl-tRNA synthetase	0.005	−0.570
gene-NATL1_19521	*rpsL*	30S ribosomal protein S12	0.001	−0.582
gene-NATL1_19871	*rpsE*	30S ribosomal protein S5	0.001	−0.605
gene-NATL1_02781	*rplJ*	50S ribosomal protein L10	0.002	−0.610
gene-NATL1_19991	*rpsS*	30S Ribosomal protein S19	0.001	−0.616
gene-NATL1_19891	*rplF*	50S ribosomal protein L6	< 0.001	−0.623
gene-NATL1_09331	*gatA*	Glutamyl-tRNA (Gln) amidotransferase A subunit	0.001	−0.631
gene-NATL1_05331	*map*	putative methionine aminopeptidase	< 0.001	−0.635
gene-NATL1_16221	*glyQ*	glycyl-tRNA synthetase, alpha subunit	0.034	−0.637
gene-NATL1_20021	*rplD*	50S ribosomal protein L4	< 0.001	−0.705
gene-NATL1_19881	*rplR*	50S ribosomal protein L18	< 0.001	−0.736
gene-NATL1_19951	*rpmC*	50S ribosomal protein L29	< 0.001	−0.752
gene-NATL1_00581	*alaS*	Alanyl-tRNA synthetase	0.002	−0.756
gene-NATL1_10481	*fmt*	putative Methionyl-tRNA formyltransferase	0.008	−0.777
gene-NATL1_17561	*tyrS*	Tyrosyl-tRNA synthetase	0.026	−0.777
gene-NATL1_03281	*pth*	Peptidyl-tRNA hydrolase	0.050	−0.785
gene-NATL1_20011	*rplW*	50S ribosomal protein L23	< 0.001	−0.791
gene-NATL1_07901	*tsf*	putative Elongation factor Ts	< 0.001	−0.821
gene-NATL1_19511	*rpsG*	30S ribosomal protein S7	< 0.001	−0.868
gene-NATL1_21311	*rplT*	50S ribosomal protein L20	< 0.001	−0.883
gene-NATL1_06131	*tdcF*	Putative translation initiation inhibitor, yjgF family	0.001	−0.939
gene-NATL1_10131	*rpsR*	30S Ribosomal protein S18	0.001	−1.140
gene-NATL1_10191	*cspR*	putative tRNA/rRNA methyltransferase (SpoU family)	0.009	−1.690

**Table 3:** List of a part of differentially expressed genes (*p* < 0.05) in low salinity acclimated *Prochlorococcus* MED4.

Gene ID	Gene name	Product	*p-*Value	log_2_FC
*Energy production and conversion*				
gene-PMM0930	*pdhB*	Pyruvate dehydrogenase E1 beta subunit	< 0.001	0.691
gene-PMM0317	*psbM*	possible Photosystem II reaction center M protein (PsbM)	0.033	0.590
gene-PMM0544	*chlB*	Light-independent protochlorophyllide reductase subunit B	0.004	0.507
gene-PMM1452	*atpH,atpD*	ATP synthase, delta (OSCP) subunit	0.042	0.476
gene-PMM0785	*prk,cbbP*	phosphoribulokinase	0.007	0.475
gene-PMM1700	*acnB*	Aconitate hydratase B	0.009	0.460
gene-PMM1454	*atpG*	ATP synthase B/B′ *CF*(0)	0.019	0.411
gene-PMM1453	*atpF*	ATP synthase B/B′ *CF*(0)	0.038	0.392
gene-PMM0223	*psbA*	Photosystem II PsbA protein (D1)	0.018	−0.427
gene-PMM1157	*psbD*	Photosystem II PsbD protein (D2)	0.008	−0.470
gene-PMM0315	*psbB*	Photosystem II PsbB protein (CP47)	0.014	−0.476
gene-PMM0461	*petA*	Cytochrome f	0.021	−0.477
gene-PMM1171	*isiB*	Flavodoxin	0.045	−0.522
gene-PMM1229	PMM1229	Dehydrogenase, E1 component	0.009	−0.577
gene-PMM0594	*ndhD*	putative NADH Dehydrogenase (complex I) subunit (chain 4)	0.001	−0.663
gene-PMM0252	*psbN*	Photosystem II reaction center N protein (psbN)	0.001	−0.919
gene-PMM0366	PMM0366	Type-1 copper (blue) domain	0.002	−0.960
gene-PMM0316	PMM0316	possible ferredoxin	< 0.001	−1.358
gene-PMM0926	*psb28*	possible Photosystem II reaction center Psb28 protein	0.041	−1.707
*Inorganic ion transport and metabolism*				
gene-PMM1032	PMM1032	ABC transporter, substrate binding protein, possibly Mn.	0.006	0.751
gene-PMM1164	*futA/afuA/idiA*	putative iron ABC transporter, substrate binding protein	< 0.001	0.950
gene-PMM0808	PMM0808	Rieske iron–sulfur protein 2Fe-2S subunit	0.019	−0.501
gene-PMM0227	*cysD*	ATP-sulfurylase	< 0.001	−0.893
gene-PMM1701	PMM1701	putative chloride channel	< 0.001	−0.984
gene-PMM0504	PMM0504	CutA1 divalent ion tolerance protein	0.004	−2.817
*Molecular chaperone*				
gene-PMM1704	*dnaK2*	Molecular chaperone DnaK2, heat shock protein hsp70-2	0.047	0.360
*Translation, ribosomal structure and biogenesis*				
gene-PMM1537	*rps13, rpsM*	30S ribosomal protein S13	0.001	1.774
gene-PMM1538	*rpmJ, rpl36*	50S Ribosomal protein L36	< 0.001	1.081
gene-PMM1688	*aspS*	Aspartyl-tRNA synthetase	0.002	0.952
gene-PMM1507	*rpsJ, rps10*	30S ribosomal protein S10	< 0.001	0.844
gene-PMM0068	*def*	putative formylmethionine deformylase	0.025	0.745
gene-PMM1661	*rpl35, rpmI*	50S ribosomal protein L35	0.006	0.724
gene-PMM1534	*rpl17, rplQ*	50S ribosomal protein L17	0.002	0.697
gene-PMM1550	*rpl29, rpmC*	50S ribosomal protein L29	0.041	0.609
gene-PMM1545	*rps8, rpsH*	30S ribosomal protein S8	0.001	0.599
gene-PMM1191	*pnp*	polyribonucleotide nucleotidyltransferase	0.003	0.592
gene-PMM0597	*thrS*	Threonyl-tRNA synthetase	0.016	0.561
gene-PMM0312	*rps1a, rpsA1*	30S ribosomal protein S1, homolog A	0.002	0.560
gene-PMM1548	*rpl14, rplN*	50S Ribosomal protein L14	0.047	0.539
gene-PMM1280	PMM1280	putative bifuntional enzyme: tRNA methyltransferase: 2-C-methyl-D-erythritol 2, 4-cyclodiphosphate synthase	0.029	0.551
gene-PMM0202	*rpl10, rplJ*	50S ribosomal protein L10	0.002	0.507
gene-PMM1662	*rpl20, rplT*	50S ribosomal protein L20	0.017	0.498
gene-PMM0870	*rpl33, rpmG*	50S Ribosomal protein L33	0.030	0.467
gene-PMM1706	*rps6, rpsF*	30S ribosomal protein S6	0.041	0.465
gene-PMM1508	*tufA*	Elongation factor Tu	0.006	0.459
gene-PMM0238	*ileS*	Isoleucyl-tRNA synthetase	0.027	0.429
gene-PMM1532	*rpl13, rplM*	50S ribosomal protein L13	0.044	0.425
gene-PMM0203	*rpl1, rplA*	50S ribosomal protein L1	0.021	0.420
gene-PMM1546	*rpl5, rplE*	50S ribosomal protein L5	0.028	0.384
gene-PMM1509	*fusA*	Elongation factor G	0.030	0.370

It has been pointed out that the response of photosystem gene expression to high salt stress might be dependent on the organism under study, based on the investigations on *Prochlorococcus* strain AS9601, *Synechocystis* PCC 6803 and *Synechococcus* PCC 7002 (Al-Hosani et al., 2015). In high salt acclimated AS9601 cells, many genes coding for components of Photosystem I, Photosystem II and chlorophyll were down-regulated. By contrast, in high salt acclimated PCC 7002, PSI genes were down-regulated but PSII genes were not changed significantly (Ludwig and Bryant, 2012). Similarly, in this study, NATL1A and MED4 also showed heterogeneity in response to low salinity stress.

### Compatible solute and transporters

Cyanobacteria generally use the salt-out strategy for salt acclimation, in which cells maintain low intracellular ion concentration and accumulate compatible solutes to establish turgor (Hagemann, 2011). Compatible solutes are low-molecular-weight organic compounds, with sucrose, glucosylglycerol (GG), glucosylglycerate (GGA) and glycine betaine (GB) being the most common ones utilized by cyanobacteria (Klähn and Hagemann, 2011). *Prochlorococcus* cells probably use GGA and sucrose as their main compatible solutes (Scanlan et al., 2009). In NATL1A, we observed significant decrease in transcript abundance of the GGA synthesis genes (*gpgP*, encoding glucosyl-phosphoglycerate phosphatase, and *gpgS*, encoding glucosyl-phosphoglycerate synthase) in the low salinity acclimated cells compared to control cells (Table 4). However, we did not observe significant change on the sucrose synthesis gene *spsA* (encoding sucrose phosphate synthase). Moreover, in MED4, all the three genes did not show significant change in transcript abundance. These results suggest that, to cope with low salinity stress, NATL1A probably reduced the concentration of intracellular compatible solute GGA, while MED4 did not reduce the concentration of the compatible solutes. Again, this different observations may be due to that the two strains were at different stress level when growing in the medium with salinity 28 psu. In another study, high salt acclimated *Prochlorococcus* AS9601 cells up-regulated the *gpgS* gene and a sodium transporter, suggesting that active extrusion of sodium ions and accumulation of GGA are involved in AS9601 acclimation to high salt stress (Al-Hosani et al., 2015). Together, these results suggests that compatible solute GGA may play an important role in the adaptation of *Prochlorococcus* to salinity changes.

**Table 4:** Expression change on genes responsible for compatible solute biosynthesis.

	NATL1A			MED4		
	Locus	*p-*Value	log_2_FC	Locus	*p*-Value	log_2_FC
*gpgP*	NATL1_05721	0.001	−0.809	PMM0515	0.367	0.214
*gpgS*	NATL1_09131	0.012	−0.431	PMM0962	0.476	−0.132
*spsA*	NATL1_21951	0.421	−0.147	PMM1711	0.143	−0.371

Na^+^/H^+^ antiporter is closely related to plant salinity tolerance, and it is one of the critical factors of plant salt tolerance. To adapt to a high salt environment, plants will reduce the plasma membrane Na^+^ level through Na^+^/H^+^ antiporter (Apse et al., 1999; Hasegawa et al., 2000). Besides, cyanobacteria cells involved in salt stress tolerance was correlated with the activity of Na^+^/H^+^ antiporter (Allakhverdiev et al., 1999, 2000). However, in this study, the transcript level of Na^+^/H^+^ antiporter (*nhaP*) was increased under low salinity stress in NATL1A cells (Table 2). It is not clear what is the mechanism involved in this phenomenon. Perhaps the increasing expression of Na^+^/H^+^ antiporter would help to reduce the cytoplasm Na^+^ level which has already adapted to high salinity level of seawater.

### Iron transporter and molecular chaperone

Interestingly, a periplasmic ABC-type Fe^3+^ transporter (*afuA/idiA/futA*) was up-regulated in low salinity acclimated cells of both NATL1A and MED4, compared to control cells. Moreover, NATL1A also up-regulated a ferric uptake regulator (NATL1_19031). It has been demonstrated that the transcript levels of *idiA* gene in *Synechococcus* PCC 6301 and *Prochlorococcus* MED4 were increased under iron deficiency conditions (Michel et al., 1999; Webb et al., 2001; Thompson et al., 2011). This suggests that cells may be iron-limited under low salt-stress. Previously, *afuA* was found to be down-regulated in high salt stressed cells of *Prochlorococcus* AS9601 (Al-Hosani et al., 2015). The authors attributed this to the reduced expression of iron required proteins under high salt condition. They also concluded that AS9601 was not iron limited because no difference in ferredoxin expression level was found between salt acclimated cells and control cells. It has been also revealed that iron requirement and siderophore production in cells is lower under high salinity (Boyle et al., 1977; Ruebsam et al., 2018). Together, these results indicates that there is a tight link between iron requirement and salt conditions in *Prochlorococcus*. However, the gene *isiB* (flavodoxin), which was induced in low iron stress (Erdner and Anderson, 1999; McKay et al., 1999), was not observed to be up-regulated in this study (Table 3). Hence, the specific relationship between low salinity stress and iron homeostasis remains to be investigated.

Up-regulated expression of *dnaK* was observed in both MED4 and NATL1A, which suggests that this gene could play a role in low salinity acclimation (Tables 2, 3). However, the molecular chaperone *dnaK* is one of the key factors for salt stress tolerance in halophiles, and over expression of *dnaK* can greatly reduce the growth lag period of the bacteria, allowing them to grow normally under salt stress (Sugimoto et al., 2003). Fukuda et al. (2001, 2002) cloned the *dnaK* gene from *Tetragenococcus halophila* JCM5888 and introduced it into *E. coli*, and found that the *dnaK* transcript abundance was increased approximately 3.5-fold under salt stress. Meanwhile, *dnaK* was also found to be present in the halotolerant cyanobacterium *Aphanothece halophytica* (Hibino et al., 1999). The gene product of *dnaK*, heat shock protein hsp70, likely plays an important role in stress resistance, no matter it is low salinity stress or high salt stress.

### Highly differentially expressed genes

When the filter criterion was changed from only meeting the value of *p* (*p* < 0.05) to meeting both value of *p* and log2fold change with values greater than 1 (high induction) or less than-1 (high inhibition), there were 81 and 30 highly differentially expressed genes in NATL1A and MED4, respectively. These number are comparable to the previous study on *Prochlorococcus* AS9601, in which 69 highly differentially expressed genes were found in high salt acclimated cells compared to control cells (Al-Hosani et al., 2015). In NATL1A, 22 genes were down-regulated and 59 were up-regulated, while in MED4, 17 genes were down-regulated and 13 were up-regulated. There was no apparent gene enrichment pattern observed among these highly differentially expressed genes (Supplementary Tables S1, S2). For example, in low-salinity stress cells of NATL1A, many genes were highly inhibited, which were related to posttranslational modification (NATL1_02111 and NATL1_13731), signal transduction mechanisms (*typA*), cell envelope biogenesis, outer membrane (NATL1_08371 and NATL1_04491), translation, ribosomal structure and biogenesis (*rpsR*), coenzyme metabolism (*folE*), energy production and conversion (*atpC*), and amino acid transport and metabolism (*proA*). Nevertheless, in salinity acclimated cells of MED4, some other genes appear to be repressed, which were those involved in energy production and conversion (PMM0316), secondary metabolites biosynthesis, transport, and catabolism (PMM0280), DNA replication, recombination, and repair (*ruvC*), lipid metabolism (*des, yocE*) and posttranslational modification (PMM1006).

## Conclusion

*Prochlorococcus* is the most abundant phototroph in the ocean. This organism has been adapted to open ocean areas with stable salt concentrations, and barely found in nearshore and estuarine waters with lower and variable salt concentrations. In this study, we showed that the lowest salinities for acclimation of high-light adapted *Prochlorococcu*s strain MED4 and low-light adapted strain NATL1A were 25 psu and 28 psu, respectively. The optimal growing salinity of both MED4 and NATL1A were from 30 to 40 psu. Global transcriptome analysis showed that the two strains responded differently to low salinity stress. First, far more genes of NATL1A were impacted than those of MED4 in low salinity acclimated cells, suggesting NATL1A was more intensively stressed than MED4 under salinity 28 psu. Second, compared to control, low salinity acclimated cells of NATL1A repressed the expression of genes involved in translation, ribosomal structure and biogenesis and ATP production, but enhanced photosynthesis, while MED4 regulated these pathways in an opposite way. To cope with low salinity, NATL1A also reduced the transcript abundance of genes involved in compatible solute GGA, while MED4 did not. Interpreting from a previous study and this study, a tight link between iron transportation and salt condition was verified, with high salinity stressed cells coupling with up-regulation of iron transporters and low salinity stressed cells coupling with down-regulation of iron transporters. This study demonstrated the regulations of global transcriptome of *Prochlorococcus* under low salinity stress and the mechanisms within those regulations warrant further investigation.

## Data availability statement

The datasets presented in this study can be found in online repositories. The names of the repository/repositories and accession number(s) can be found below: https://www.ncbi.nlm.nih.gov/geo/query/acc.cgi?acc=GSE195946.

## Author contributions

SH, JD, and LL designed the experiments. XH and HL performed the experiments and analyzed the data. SH and XH wrote the manuscript. JD and LL provided resources and supervision. All authors contributed to the article and approved the submitted version.

## Funding

This study was funded by the National Natural Science Foundation of China [grant number 42176116, 41576126]; Key Special Project for Introduced Talents Team of Southern Marine Science and Engineering Guangdong Laboratory (Guangzhou) [GML2019ZD0404]; the Natural Science Foundation of Guangdong Province [grant number 2017A030306020]; the Youth Innovation Promotion Association [grant number 2018377]; and the Rising Star Foundation of the South China Sea Institute of Oceanology [grant number NHXX2019ST0101].

## Conflict of interest

The authors declare that the research was conducted in the absence of any commercial or financial relationships that could be construed as a potential conflict of interest.

## Publisher’s note

All claims expressed in this article are solely those of the authors and do not necessarily represent those of their affiliated organizations, or those of the publisher, the editors and the reviewers. Any product that may be evaluated in this article, or claim that may be made by its manufacturer, is not guaranteed or endorsed by the publisher.

## Footnotes

^1^ https://chisholmlab.mit.edu
