# Supplementary material for: Acclimation and stress response of Prochlorococcus to low salinity
Source: Front Microbiol. 2022 Oct 13;13:1038136. doi: 10.3389/fmicb.2022.1038136 (PMC9606707; doi:10.3389/fmicb.2022.1038136)
Supplement: Supplementary file 1 [file Data_Sheet_1.docx]

**Supplementary material**

**Acclimation and stress response of *Prochlorococcus* to low salinity**

Xiayu He^1,2^, Huan Liu^1,2,^ Lijuan Long^1,3^, Junde Dong^1,3^, Sijun Huang^1,3*^

1. Key Laboratory of Tropical Marine Bio-resources and Ecology, South China Sea Institute of Oceanology, Chinese Academy of Sciences, Guangzhou 510301
2. University of Chinese Academy of Sciences, Beijing 100049
3. Southern Marine Science and Engineering Guangdong Laboratory (Guangdong), Guangzhou 511458

^*^Corresponding author: huangsijun@scsio.ac.cn

**Table S1. Highly differentially expressed genes in low salinity acclimated cells of NATL1A using a false discovery rate (*p*-value < 0.05) and log_2_fold change values > 1 and < -1**

| **Gene Name** | **Definition** | **Cyanobase functional category** | ***p*-value** | **Log_2_FC** | **Regulation** |
| --- | --- | --- | --- | --- | --- |
| NATL1_12621 | Hypothetical protein | Not in COGs | < 0.001 | 2.627 | Up |
| *wza* | Hypothetical protein | Cell wall/membrane/envelope biogenesis | < 0.001 | 2.444 | Up |
| NATL1_13941 | Hypothetical protein | Not in COGs | < 0.001 | 2.103 | Up |
| NATL1_08591 | Nucleotide-diphosphate-sugar epimerase,membrane associated | Cell envelope biogenesis, outer membrane / Carbohydrate transport and metabolism | < 0.001 | 2.012 | Up |
| NATL1_08811 | Hypothetical protein | Not in COGs | < 0.001 | 1.962 | Up |
| NATL1_17971 | Hypothetical protein | Not in COGs | < 0.001 | 1.904 | Up |
| *wecC* | UDP-glucose 6-dehydrogenase | Cell envelope biogenesis, outer membrane | < 0.001 | 1.704 | Up |
| NATL1_12121 | Hypothetical protein | Not in COGs | < 0.001 | 1.593 | Up |
| *ctaA* | Uncharacterized protein required for cytochrome oxidase assembly | Posttranslational modification, protein turnover, chaperones | < 0.001 | 1.573 | Up |
| NATL1_18081 | Hypothetical protein | Not in COGs | < 0.001 | 1.569 | Up |
| NATL1_08671 | Hypothetical protein | General function prediction only | 0.002 | 1.569 | Up |
| NATL1_12981 | Hypothetical protein | Not in COGs | < 0.001 | 1.539 | Up |
| NATL1_12321 | Hypothetical protein | Not in COGs | < 0.001 | 1.529 | Up |
| NATL1_04471 | Uncharacterized protein conserved in bacteria | Function unknown | < 0.001 | 1.501 | Up |
| *dnaJ2* | DnaJ2 protein | Posttranslational modification, protein turnover, chaperones | 0.001 | 1.481 | Up |
| NATL1_11331 | Hypothetical protein | Not in COGs | < 0.001 | 1.448 | Up |
| NATL1_02971 | conserved hypothetical | Not in COGs | < 0.001 | 1.412 | Up |
| NATL1_00911 | Hypothetical protein | Not in COGs | < 0.001 | 1.398 | Up |
| NATL1_11931 | Hypothetical protein | Not in COGs | < 0.001 | 1.395 | Up |
| *pabC* | Aminotransferases class-IV | Amino acid transport and metabolism / Coenzyme metabolism | 0.004 | 1.391 | Up |
| NATL1_14171 | Hypothetical protein | Not in COGs | < 0.001 | 1.387 | Up |
| NATL1_11851 | Hypothetical protein | Not in COGs | < 0.001 | 1.367 | Up |
| NATL1_18181 | Hypothetical protein | Not in COGs | < 0.001 | 1.365 | Up |
| NATL1_12331 | Hypothetical protein | Not in COGs | < 0.001 | 1.356 | Up |
| NATL1_12231 | Hypothetical protein | Not in COGs | < 0.001 | 1.338 | Up |
| NATL1_05531 | Putative principal RNA polymerase sigma factor | Transcription | < 0.001 | 1.323 | Up |
| NATL1_00901 | Hypothetical protein | Not in COGs | 0.004 | 1.322 | Up |
| *proC* | Delta 1-pyrroline-5-carboxylate reductase | Amino acid transport and metabolism | < 0.001 | 1.321 | Up |
| NATL1_15531 | Hypothetical protein | Not in COGs | < 0.001 | 1.308 | Up |
| NATL1_00521 | conserved hypothetical protein | Not in COGs | < 0.001 | 1.273 | Up |
| NATL1_21371 | conserved hypothetical protein | Not in COGs | < 0.001 | 1.261 | Up |
| NATL1_06001 | conserved hypothetical | Not in COGs | < 0.001 | 1.257 | Up |
| *cyoA* | putative cytochrome c oxidase,subunit 2 | Energy production and conversion | < 0.001 | 1.249 | Up |
| NATL1_19071 | Cobalamin synthesis protein/P47K | General function prediction only | < 0.001 | 1.232 | Up |
| NATL1_01541 | conserved hypothetical protein | Not in COGs | < 0.001 | 1.230 | Up |
| *cyoE* | putative protoheme IX farnesyltransferase | Posttranslational modification, protein turnover, chaperones | < 0.001 | 1.226 | Up |
| NATL1_20301 | conserved hypothetical protein | Not in COGs | < 0.001 | 1.224 | Up |
| *afuA* | putative iron ABC transporter,substrate binding protein | Inorganic ion transport and metabolism | < 0.001 | 1.208 | Up |
| NATL1_00991 | Hypothetical protein | Not in COGs | < 0.001 | 1.207 | Up |
| NATL1_01431 | possible 4'-phosphopantetheinyl transferase family protein | Coenzyme metabolism | 0.005 | 1.203 | Up |
| NATL1_18261 | Hypothetical protein | Not in COGs | < 0.001 | 1.203 | Up |
| NATL1_20641 | Conserved hypothetical protein | Not in COGs | 0.001 | 1.168 | Up |
| NATL1_09701 | Predicted transcriptional regulator containing the HTH domain | DNA replication, recombination and repair | < 0.001 | 1.135 | Up |
| NATL1_08711 | Hypothetical protein | Not in COGs | < 0.001 | 1.132 | Up |
| NATL1_00461 | Hypothetical protein | Not in COGs | 0.001 | 1.123 | Up |
| NATL1_01031 | Hypothetical protein | Not in COGs | < 0.001 | 1.101 | Up |
| NATL1_01091 | Hypothetical protein | Cell division and chromosome partitioning | < 0.001 | 1.100 | Up |
| *aroQ* | Dehydroquinase class II | Amino acid transport and metabolism | 0.001 | 1.094 | Up |
| NATL1_16211 | porin-like protein | Not in COGs | < 0.001 | 1.074 | Up |
| NATL1_21781 | Type II alternative RNA polymerase sigma factor,sigma-70 family | Transcription | < 0.001 | 1.047 | Up |
| NATL1_12791 | Hypothetical protein | Not in COGs | < 0.001 | 1.046 | Up |
| *mscS* | small mechanosensitive ion channel,MscS family | Cell envelope biogenesis, outer membrane | < 0.001 | 1.045 | Up |
| NATL1_11091 | Hypothetical protein | DNA replication, recombination, and repair | 0.001 | 1.037 | Up |
| *miaE* | putative tRNA-(MS[2]IO[6]A)-hydroxylase-like protein | Nucleotide transport and metabolism / Translation, ribosomal structure and biogenesis | < 0.001 | 1.030 | Up |
| NATL1_15471 | Hypothetical protein | Not in COGs | < 0.001 | 1.027 | Up |
| *tal* | Transaldolase | Carbohydrate transport and metabolism | < 0.001 | 1.024 | Up |
| NATL1_19061 | Hypothetical protein | Not in COGs | < 0.001 | 1.014 | Up |
| NATL1_21411 | conserved hypothetical protein | Not in COGs | < 0.001 | 1.013 | Up |
| *cobS* | Cobalamin-5-phosphate synthase CobS | Coenzyme metabolism | < 0.001 | 1.012 | Up |
| *dedA* | DedA family; putative alkaline phosphatase-like protein | Function unknown | < 0.001 | -1.012 | Down |
| *proA* | Gamma-glutamyl phosphate reductase | Amino acid transport and metabolism | < 0.001 | -1.018 | Down |
| *atpC* | ATP synthase,Epsilon subunit | Energy production and conversion | < 0.001 | -1.089 | Down |
| *folE* | GTP cyclohydrolase I | Coenzyme metabolism | < 0.001 | -1.110 | Down |
| NATL1_18211 | Hypothetical protein | General function prediction only | 0.001 | -1.112 | Down |
| *rpsR* | 30S Ribosomal protein S18 | Translation, ribosomal structure and biogenesis | 0.001 | -1.140 | Down |
| NATL1_07911 | possible Adenylate cyclase | Not in COGs | 0.002 | -1.170 | Down |
| *sfsA* | putative sugar fermentation stimulation protein | General function prediction only | 0.001 | -1.184 | Down |
| NATL1_04491 | possible Glycosyl transferase,group 1 | Cell envelope biogenesis, outer membrane | < 0.001 | -1.191 | Down |
| NATL1_19661 | Hypothetical protein | Not in COGs | < 0.001 | -1.221 | Down |
| NATL1_08371 | possible mRNA binding protein | Cell envelope biogenesis, outer membrane / Carbohydrate transport and metabolism | < 0.001 | -1.266 | Down |
| NATL1_13741 | Hypothetical protein | Not in COGs | 0.005 | -1.277 | Down |
| *typA* | tyrosine binding protein | Signal transduction mechanisms | < 0.001 | -1.325 | Down |
| NATL1_09171 | Hypothetical protein | Not in COGs | 0.001 | -1.355 | Down |
| NATL1_14411 | Hypothetical protein | Not in COGs | < 0.001 | -1.378 | Down |
| NATL1_08021 | possible permease | General function prediction only | 0.001 | -1.381 | Down |
| NATL1_06431 | conserved hypothetical | Not in COGs | 0.003 | -1.411 | Down |
| NATL1_13731 | Hypothetical protein | Posttranslational modification, protein turnover, chaperones | < 0.001 | -1.479 | Down |
| NATL1_18471 | Hypothetical protein | Not in COGs | < 0.001 | -1.500 | Down |
| NATL1_05681 | Hypothetical protein | Not in COGs | 0.001 | -1.626 | Down |
| NATL1_06171 | possible acetyltransferase | General function prediction only | < 0.001 | -1.871 | Down |
| NATL1_02111 | putative molecular chaperone | Posttranslational modification, protein turnover, chaperones | < 0.001 | -2.414 | Down |

**Table S2. Highly differentially expressed genes in low salinity acclimated cells of MED4 using a false discovery rate (*p*-value < 0.05) and log_2_fold change values > 1 and < -1**

| **Gene Name** | | **Definition** | | **Cyanobase functional category** | ***p*-value** | **logFC** | **Regulation** |
| --- | --- | --- | --- | --- | --- | --- | --- |
| PMM0814 | | Possible Cytochrome oxidase c subunit VIb | | Not in COGs | < 0.001 | 1.927 | Up |
| rps13,rpsM | | 30S ribosomal protein S13 | | Translation, ribosomal structure and biogenesis | 0.001 | 1.774 | Up |
| PMM0348 | | Possible Spectrin repeat | | Not in COGs | < 0.001 | 1.754 | Up |
| PMM0087 | | Conserved hypothetical protein | | Not in COGs | < 0.001 | 1.601 | Up |
| PMM2013 | | Hypothetical protein | | Not in COGs | 0.001 | 1.206 | Up |
| PMM1602 | | Conserved hypothetical protein | | Not in COGs | < 0.001 | 1.090 | Up |
| rpmJ,rpl36 | | 50S Ribosomal protein L36 | | Translation, ribosomal structure and biogenesis | < 0.001 | 1.081 | Up |
| PMM1881 | | Conserved hypothetical protein | | Not in COGs | < 0.001 | 1.071 | Up |
| PMM1429 | | Conserved hypothetical protein | | Not in COGs | < 0.001 | 1.067 | Up |
| *secE* | | Putative preprotein translocase, SecE subunit | | Not in COGs | < 0.001 | 1.057 | Up |
| PMM0596 | | Putative glucokinase | | Carbohydrate transport and metabolism | 0.002 | 1.039 | Up |
| PMM1400 | | Possible Hemagglutinin-neuraminidase | | Not in COGs | < 0.001 | 1.039 | Up |
| *rnhA* | | Possible ribonuclease HI | | DNA replication, recombination, and repair | 0.001 | 1.033 | Up |
| PMM1006 | | Glutathione peroxidase | | Posttranslational modification, protein turnover, chaperones | < 0.001 | -1.041 | Down |
| *des,yocE* | | Fatty acid desaturase,type 2 | | Lipid metabolism | < 0.001 | -1.062 | Down |
| *pgk,cbbK* | | Phosphoglycerate kinase | | Carbohydrate transport and metabolism | < 0.001 | -1.119 | Down |
| PMM2051 | | Hypothetical protein | | Not in COGs | < 0.001 | -1.140 | Down |
| *ruvC* | Crossover junction endodeoxyribonuclease RuvC | | | DNA replication, recombination, and repair | < 0.001 | -1.178 | Down |
| PMM0280 | | Retinal pigment epithelial membrane protein | | Secondary metabolites biosynthesis, transport, and catabolism | < 0.001 | -1.180 | Down |
| *ffs* | | Signal recognition particle RNA (4.5 S RNA) | | Not in COGs | < 0.001 | -1.252 | Down |
| PMM1028 | | Conserved hypothetical | | Not in COGs | 0.001 | -1.318 | Down |
| PMED4_ncRNA_Yfr11 | | | - | Not in COGs | < 0.001 | -1.335 | Down |
| PMM0316 | | Possible ferredoxin | | Energy production and conversion | < 0.001 | -1.358 | Down |
| PMM1359 | | Conserved hypothetical protein | | Function unknown | < 0.001 | -1.413 | Down |
| PMM1894 | | Conserved hypothetical protein | | Not in COGs | < 0.001 | -1.520 | Down |
| PMM1854 | | Conserved hypothetical protein | | Not in COGs | 0.001 | -1.584 | Down |
| tRNA-Gly1 | | tRNA-Gly | | Not in COGs | < 0.001 | -1.990 | Down |
| PMM1961 | | Conserved hypothetical protein | | Not in COGs | 0.001 | -2.139 | Down |
| PMM2003 | | Conserved hypothetical protein | | Not in COGs | < 0.001 | -2.187 | Down |
| PMM0731 | | Possible COMC family | | Not in COGs | < 0.001 | -3.506 | Down |
